# Digital Outpatient Services for Adults: Development of an Intervention and Protocol for a Multicenter Non–Randomized Controlled Trial

**DOI:** 10.2196/46649

**Published:** 2023-07-10

**Authors:** Heidi Holmen, Are Martin Holm, Thomas Karsten Kilvær, Tone Marte Ljoså, Lars-Petter Granan, Christopher Ekholdt, Lotte Sandberg Larsen, Erik Fosse

**Affiliations:** 1 Intervention Centre Division of Technology and Innovation Oslo University Hospital Oslo Norway; 2 Department of Nursing and Health Promotion Faculty of Health Sciences Oslo Metropolitan University Oslo Norway; 3 Department of Respiratory Diseases Division of Cardiovascular and Pulmonary Diseases Oslo University Hospital Oslo Norway; 4 Institute for Clinical Medicine Faculty of Medicine University of Oslo Oslo Norway; 5 Department of Cancer University Hospital of Northern Norway Tromsø Norway; 6 Institute for Clinical Medicine Faculty of Medicine The Arctic University of Norway Tromsø Norway; 7 Department of Pain Management and Research Division of Emergencies and Critical Care Oslo University Hospital Oslo Norway; 8 Division of Clinical Neuroscience Oslo University Hospital Oslo Norway

**Keywords:** cancer, complex pain, digital solution, epilepsy, health literacy, interstitial lung disease, mHealth, outpatient, patient-reported outcome measures, remote monitoring, self-monitoring

## Abstract

**Background:**

Health care services are being challenged by an increasing number of patients and limited resources. Hence, research investigating options to reduce costs and increase effectiveness is warranted. Digital outpatient services can provide flexible and tailored follow-up, improve patients’ health literacy, and facilitate the identification of adverse courses of disease. However, previous research largely focused on disease-specific contexts and outcomes. Therefore, research on digital services investigating generic outcomes such as health literacy is warranted.

**Objective:**

This article aims to describe the “digital outpatient service” intervention and present the protocol for an ongoing multicenter, nonrandomized trial evaluating this intervention.

**Methods:**

Based on previous experiences and evidence-based knowledge, we developed this intervention through patient-journey maps in collaboration with each clinical specialty. The patients gain access to a mobile app for self-monitoring and patient-reported outcomes and a chat for contact between the patients and health care workers. The health care workers’ dashboard includes a traffic light system to draw attention to the most urgent patient reports. In this multicenter, non–randomized controlled trial, patients are allocated to the control group receiving standard care or the 6-month intervention. Eligible patients are aged 18 years or older who receive outpatient care at the neurology, lung, pain, or cancer departments at 2 university hospitals in Norway. Our evaluation will include patient-reported outcomes, qualitative interviews, and clinical measures. The primary outcome will be health literacy using the Health Literacy Questionnaire. A sample size of 165 participants is split into a 1:2 ratio in favor of the intervention. We will analyze quantitative data in SPSS (IBM Corp) using descriptive statistics and logistic regression, and qualitative data using thematic analysis.

**Results:**

This trial started in September 2021, and the intervention started in January 2022. Recruitment has ended, with 55 patients in the control group and 107 patients in the intervention group. Follow-up is expected to end in July 2023, with results expected to be obtained in December 2023.

**Conclusions:**

This study will evaluate an intervention facilitated by an already certified digital multicomponent solution, with intervention content based on patient-reported outcomes, health literacy, and self-monitoring. The intervention is specifically tailored to each participating center and the needs of their patients using patient journey maps. The comprehensive and generic evaluation of this digital outpatient service intervention is a strength as it targets a heterogeneous sample of patients. Thus, this study will provide important knowledge about the applicability and effects of digital health care services. As a result, patients and health care workers will gain a new, evidence-based understanding of whether and how digital tools may be used in clinical care.

**Trial Registration:**

ClinicalTrials.gov NCT05068869; https://clinicaltrials.gov/ct2/show/NCT05068869

**International Registered Report Identifier (IRRID):**

DERR1-10.2196/46649

## Introduction

While advanced medicine contributes to prolonged life expectancy, the number of patients increases, and health service resources remain limited [1]. The reduction in hospital overnight capacity often increases the number of outpatient consultations. There is a risk of suboptimal resource use in current health services as these do not sufficiently meet individuals’ needs for understandable health information [2,3]. Digital health solutions, which have emerged over the last decades, are embraced and called for by health authorities [4,5], and the recent COVID-19 pandemic has led to a fast, large-scale adoption [6]. Systematic reviews provide some evidence about the effects of digital solutions [7-9], but the extent to which digital services can improve patient outcomes or resource use has not been firmly established [10].

Any implementation of digital health solutions requires patients’ and health care workers’ understanding of why and how the digital possibilities are used [11,12]. Thus, it is necessary to attain a certain level of health literacy, defined as “the cognitive and social skills that determine the motivation and ability of individuals to gain access to, understand, and use information in ways which promote and maintain good health” [13]. Health literacy is closely related to patients’ self-management and decisions related to their health [14], and eHealth literacy is linked to the use of digital solutions for health [12]. Low health literacy is associated with poorer health outcomes [15], lower self-management, and less use of digital health solutions [2,16]. It is reasonable to expect that better health literacy will improve self-management and the ability to benefit from digital health solutions [12]. The paucity of systematic reviews supports research focusing on the possibilities of digital interventions on health literacy [12,16].

Digital health solutions have embraced the use of patient-reported outcome measures (PROMs), allowing patients to subjectively report their health, pain, symptoms, and other relevant parameters [17]. PROMs allow health care workers to individualize patient care, although it has been challenging to demonstrate clinical effects in research [18]. However, effect evaluations should be part of studies on how PROMs are used in a clinical setting, and explanatory factors should be identified [19-21]. When routinely used, PROMs may support self-management and communication between patients and health care workers [17,19].

Allowing patients to digitally engage, self-monitor, and share data with health care workers has some obvious advantages and challenges [16,20,22,23]. From both the patient’s and the health care worker’s perspectives’, digital solutions must be usable, clinically relevant, convenient, and evidence based. Patients rely on health care workers to assess their data; likewise, health care workers depend on patients to report the assigned health parameters without under- or overreporting symptoms [24]. Digital solutions in outpatient care may contribute to the prevention of complications or exacerbations by promoting contacts between patients and health care workers, allowing the latter to intervene earlier and to act according to clinical needs [25]. A recent systematic review found that using digital solutions increased patients’ engagement in the technical usability of the solutions that affected their everyday lives [26]. Patients were also found to report more confidence in and knowledge of their own conditions and increased autonomy. These findings support those of earlier research on patients’ engagement and the impacts of digital solutions [27].

There is limited research regarding the development and impacts of multicomponent digital solutions that include PROMs, remote monitoring, patient notifications, alerts for health care workers, asynchronous chats, and video consultations. An evaluation of a heterogenous range of digital health interventions found some positive effects on coping, quality of life, and pain in cancer treatment [28], as well as alleviation of both pain and functional disabilities in disorders associated with musculoskeletal pain [29]. Primary research in home monitoring of symptoms enables detection of exacerbations and progression in patients with interstitial lung disease (ILD) [30], but there is a need for evidence to support new ILD interventions [31]. There has been wide adoption and positive effects of mobile apps for patients with epilepsy, although less so in collaboration with health care workers [32]. The need to study homogeneous, static, and standardized interventions with a high level of fidelity [33] might explain why research on digital solutions is rarely conducted despite the frequency of their application in clinical care. Furthermore, clinical challenges remain, particularly regarding the integration of electronic health records into existing platforms [22]. There is limited knowledge about how digital systems fit current workflows and about how data and PROMs should be standardized to best generate registry data of value for other clinical sites and researchers [16,23]. Altogether, there is a paucity of empirical studies on the implementation and impacts of digital health solutions on outpatient care.

The purposes of this article are to (1) describe the digital outpatient service intervention and how it promotes digital outpatient care, and (2) present a multimethod protocol for a multicenter, nonrandomized trial to evaluate the intervention.

## Methods

In the following, the development of the digital outpatient service intervention is presented before the details of the planned trial. The aim of the planned trial is to evaluate whether this intervention can have positive impacts on outcomes, including health literacy, health-related quality of life (HRQL), digital health literacy, satisfaction, and use of health service resources. Qualitative interviews will provide in-depth knowledge about the intervention from the perspectives of patients and health care workers. Furthermore, possible care pathways that ensure the quality of care and efficiency of remote monitoring in digital outpatient care will be investigated. The reporting of the intervention was guided by the Template for Intervention Description and Replication (TiDiER) [34], the protocol is reported according to the SPIRIT (Standard Protocol Items: Recommendations for Interventional Trials) [35], and the PROMs are reported according to the SPIRIT PRO extension [36].

### Development of the Digital Outpatient Service Intervention

#### Participants in the Development of the Intervention

The intervention was developed through collaboration among researchers from the Oslo University Hospital (OUH) intervention center, product managers at Dignio Connected Care, and health care workers and health care researchers from each participating department. These include the Department of Respiratory Diseases, the Department of Neurology, and the Department of Pain Management at OUH, as well as the Department of Cancer at the University Hospital of North Norway (UNN). To achieve an intervention suitable for patients’ needs and available staffing resources, each department tailored the intervention to their patient group and organizational structure.

#### Patient and Public Involvement

Representatives from the Norwegian Cancer Society contributed to the project’s development. Furthermore, the intervention for the neurology department is based on content developed together with patients and stakeholders. Health care workers are included as they constitute significant users in this project. The MyDignio app has been through several patient reviews before the current version.

#### Essential Elements and Aim of the Intervention

At the core of the intervention in all 4 departments is increased access to outpatient services. On the digital platform, patients can respond to PROMs and self-monitor parameters relevant to their conditions and have asynchronous, easy-access contact with health care workers. Designated health care staff will assess these data and act accordingly. These elements may facilitate flexible patient follow-up, building on a conceptual model that explains associations between health literacy and health outcomes, such as access to services, use of services, interactions between patients and health care workers, and patient self-care [37]. Patients can have an increased influence on their care and their contact with health care services through self-reported subjective experiences related to their care needs and their wishes for follow-up, in combination with their reports of objective measures such as blood pressure or oxygen saturation. This may facilitate timely contacts between patients and health care workers, where patients can receive guidance based on their questions, and likewise enable health care workers to identify patient struggles more accurately.

#### The Digital Platform for the Intervention: Dignio Connected Care

The platform for the digital outpatient service intervention is Dignio Connected Care [38], consisting of the multicomponent cloud-based system Dignio Prevent for health care workers and the MyDignio patient app. It is CE (Conformité Européenne: French for “European conformity”) marked, satisfies all regulatory requirements for privacy and information security, and has been used in various clinical settings in Norway [39-41], the United Kingdom [42], and China [38]. The digital platform can be tailored to the needs of individual patients in consultation with their health care workers. Components can be added to individualize follow-ups (Tables 1 and 2) and will vary depending on the treatment at any given time. Examples of the interface are given in the Multimedia Appendices 1 and 2.

**Table 1:** Patient-reported components used in the digital outpatient service.

Dignio component			Short description		Center using the component in its intervention	
Patient-reported outcome measures (PROMs)			Standardized and individualized PROMs with numerical scales, single and multiple-choice answers, and free text.		Cancer: Edmonton Symptom Assessment System (ESAS-r) [43], Eastern Cooperative Oncology Group (ECOG) scale assessing functional level [44], PROM version of the Common Terminology Criteria for Adverse Events (PRO-CTCAE) [45] and items on side effects of chemotherapy. The frequency of administration is per individual needs. Lung: King’s Brief Interstitial Lung Disease questionnaire (K-BILD) [46] assessing self-reported health every 12th week, and items on side effects (vomiting, rash, dizziness, abdominal pain, or diarrhea), administered every 4th week. Neuro: PRO-EPI: A multidimensional epilepsy PROM with items on seizures, medication, living with epilepsy, and need for health services developed by clinicians and the Norwegian Epilepsy Network for health care workers [47,48], administered every 11th week. Pain: Items on pain on a visual analog scale, intensity, location, side effects (constipation, sweat, anxiety, etc), compliance with current treatment and need for health services, administered per medication plan when changes to this plan are made.	
**Physiologic measures**						
	Blood pressure	Bluetooth devices or BYOD^a^		Cancer, on clinical indication		
	Body temperature	Bluetooth devices or BYOD		Cancer, on clinical indication		
	Body weight	Bluetooth devices or BYOD		Lung, weekly		
	Spirometry values	Bluetooth device		Lung, weekly		
	Oxygen saturation	Bluetooth device		Lung, weekly		
	Pulse	Bluetooth devices or BYOD		Lung, weekly		

^a^BYOD: bring your own device.

**Table 2:** Functionality for remote monitoring and communication.

Dignio component			Brief description
Tasks			Health care workers can specify tasks for patients, such as performing daily activities, registering measures taken with the patients’ own devices, or watching patient videos.
Thresholds			Health care workers can define thresholds. The limit values are set, based on each patient’s baseline, and follows the traffic light model, with green, yellow, and red alerts.
Notifications and triage			When measures are outside defined thresholds, health care workers are notified. Deviating values, responses, and unanswered messages from patients are captured by an overview of patients in Dignio Prevent, where they are sorted by degree of urgency or severity (traffic light model). If the patient does not perform the defined tasks at the specified time, a warning is triggered. This can admittedly be turned off if desired.
Reminders			Reminders can be sent to the patients as push-notifications in MyDignio, through email or SMS texts. When the task or measure is done, the reminders stop.
Messages or chat			Patients and health care workers can send messages to each other and have an asynchronous dialogue when it is best for both parties. Lung: patients are asked to upload photos of the results of any laboratory tests conducted and the results of a 6-minute walking test conducted at their local hospital every 12th week. Neuro: patients are allowed to upload photos of seizures whenever relevant.
Video consultations			A video consultation can be arranged if needed. A video room is created with an encrypted connection where the patient and health care worker can meet.
Information pages			MyDignio has an information page that can be updated by health care workers from the web portal Dignio Prevent. It is possible to create an individual information page, create templates, and send updates to several patients.
**Efficacy tools (templates)**			
	Templates for patient care	Templates can be developed to facilitate a standardized follow-up at given times, allowing health care workers to monitor any changes in a timely manner.	

#### Patient Journey Maps

**Figure 1:**
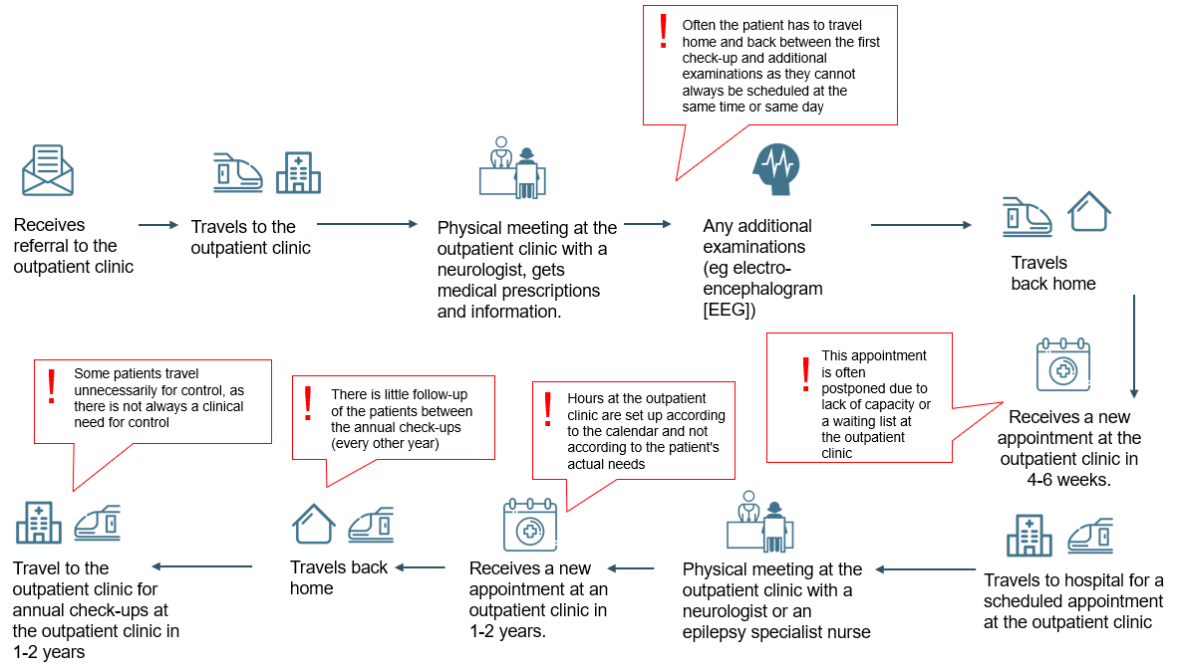
Patient journey map from the neurology department: as-is.

**Figure 2:**
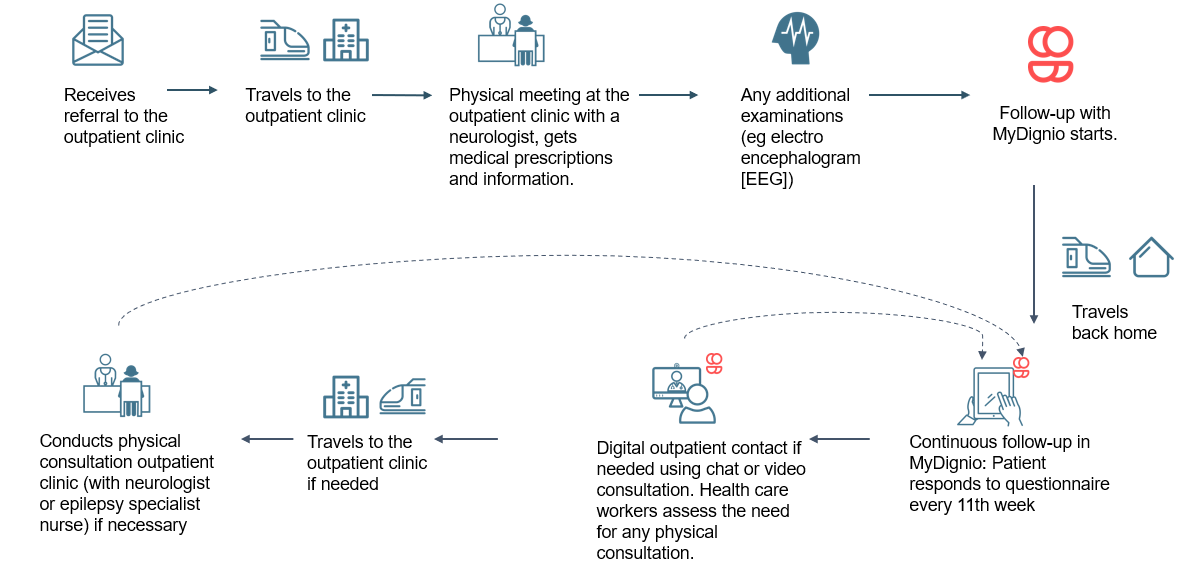
Patient journey map from the neurology department: to-be.

In collaboration with each department, patient journey maps have been drawn [49] to target the potential for a digital outpatient service and assess how the use of digital tools may affect patient outcomes, patient flow, and health care workflow. Common challenges, visualized through the red exclamation marks (Figures 1 and 2), include limited health care worker resources, an overwhelming number of patients, time-consuming telephone calls, and the challenge of contacting patients at risk (see Multimedia Appendix 3 for further examples). Patients may have a limited overview of their parameters, including PROMs and clinical data, compromising their health literacy and self-monitoring.

#### Information and Training

The patients receive a brief introduction and digital user manuals for the app. Patients using technical equipment such as spirometers are instructed to ensure correct technique and synchronization with MyDignio. Patients in the intervention group can contact their health care worker or a researcher for technical inquiries.

Most of the health care workers are familiar with the platform as they are involved in the development of the individual interventions at each center. All new health care workers receive training by the Dignio personnel or the assigned administrator at their center, and the training will be repeated upon request. Training in interpreting scores reported by the patients is provided within each department based on the needs of the health care workers. For the majority of the involved health care personnel, the PROMs applied are already used paper-based in the clinics; thus, a dialogue between the programmers from Dignio Connected Care and the health care workers has facilitated the use of already standardized cutoffs.

Implementation is secured through the involvement of dedicated health care workers within each department that contributed to the development of the intervention, the summarizing of the “as-is,” and the potential and suggestions of the “to-be” that had received training in the digital platform. In addition, designated health care workers were compensated for 10%-20% of their time to contribute to the intervention.

### Study Design for the Planned Trial to Evaluate the Intervention

**Figure 3:**
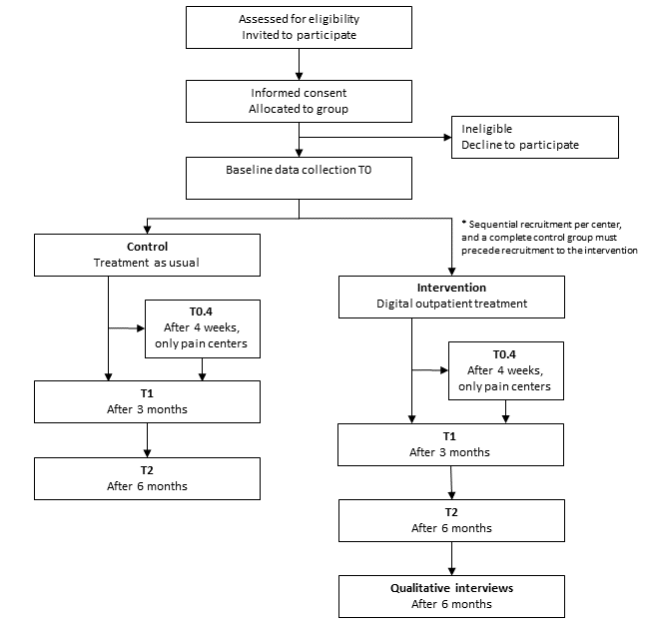
Flowchart of the multicenter non–randomized controlled trial.

The study is a multicenter, non–randomized controlled trial with 2 treatment arms and a 6-month follow-up (Figure 3). The comprehensive evaluation is based on the method for assessment of telemedicine (MAST) [50], including elements regarding the purpose and maturity of the technology, how the health problems align with the technology, safety, clinical effectiveness, patient perspectives, economic aspects, organizational, and sociocultural, ethical, and legal aspects, followed by an assessment of cross border between countries, transferability, and generalizability to other contexts. The relevance of MAST lies in its thorough assessment of the preceding conditions and its multidisciplinary assessments. Patients are currently allocated to 1 of the 2 arms. At each department, recruitment to the control arm will be completed before inclusion in the intervention arm. The control arm will receive follow-up *per the* routines at each department, largely as described in the patient journey maps labeled “as-is” (Figures 1 and 2, and Multimedia Appendix 3). The intervention arm will receive our digital outpatient service. Quantitative measures are collected longitudinally, and qualitative interviews are conducted postintervention.

### Patient Participants and Recruitment

Patients at the Department of Respiratory Diseases, the Department of Neurology, and the Department of Pain Management at OUH, as well as the Department of Cancer at UNN, are eligible for participation in the study if they are 18 years of age or older, home-based, and able to fill out Norwegian questionnaires (Table 3). Both newly diagnosed or newly referred patients, those with a diagnostic history, and those already in outpatient care are eligible for inclusion.

Eligible patients are identified through an outpatient consultation or through patient lists. All patients receive written and oral information about the project from a nurse or physician before giving their consent. The patients are not regarded as consenting participants until they have signed the consent form and filled out the baseline questionnaire. To reduce contamination in the intervention arm, patients must consent and fill out the baseline questionnaire before accessing the digital intervention.

The recruitment of patients to the qualitative interviews after the intervention is based on their consent to receive information regarding the interviews. Purposive sampling is applied to reach a heterogeneous sample with a balanced diversity in age, gender, and use of the intervention. Thus, in-depth knowledge can be obtained from both high and low users.

**Table 3:** Eligibility criteria for patients.

Patient groups	Inclusion criteria	Exclusion criteria
Patients with cancer	Cancer Receiving active treatment	Life expectancy: less than 6 months Expected need for follow-up: less than 6 months
Patients with interstitial lung disease (ILD)	ILD	Very low degree of function with risk of cognitive influence
Patients with epilepsy	Epilepsy	Complex causes of epilepsy Severe comorbidity Other planned or ongoing assessment and treatment, including assessment of unexplained illness or testing of medication
Patients with long-term, complex pain	Drug testing, adjustment, and dose reduction Self-administering medication	Comorbidity that directly affects the drug adjustment Interference with other ongoing treatment Cognitive impairment Not living at home

### Health Care Personnel Participants

Health care workers in the hospital departments are eligible for the qualitative interviews if they are affiliated with the included departments and have had a role in this project. This group includes leaders and health care workers, such as nurses and physicians, with hands-on management of patient follow-up through the intervention.

### Randomization and Blinding

This is a non–randomized controlled trial without blinding. Both patients and health care workers are familiar with the treatment arm to which the participants belong during the study.

### Outcomes to be Measured

#### Overview

The study will consider the elements affected by both external factors in the health care services and internal factors held by the patient. Patients will self-report on the questionnaires (Tables 4 and 5). Clinical parameters and information on the use of resources will be collected from electronic medical records. The primary outcome is the health literacy questionnaire (HLQ) domain 9 “understanding health information well enough to know what to do” [51]. This domain encompasses health actions relevant for a digital service, such as basic reading and understanding of health information, following instructions from health care workers, and the ability to fill out forms correctly. The secondary outcomes are health literacy, eHealth literacy, HRQL, and acceptability of the digital intervention (Table 4).

**Table 4:** Outcome measures for health literacy, health-related quality of life, digital health literacy, satisfaction, and COVID-19.

Measures, domains, and variables				Response options		Scale		Interpretation		Time point				
										T0		T1		T2
**Health literacy**														
	**Health literacy questionnaire (HLQ) (5 of 9 domains)**													
		Feeling understood and supported by health care providers (4 items) Having sufficient information to manage my own health (4 items) Actively managing my own health (5 items)	All items have the same response options: strongly disagree, disagree, agree, or strongly agree		1-4		A higher score indicates a higher level of health literacy		✓		✓		✓	
		Ability to actively engage with health care providers (5 items) Understanding health information well enough to know what to do (5 items)	All items have the same response options: cannot do or always difficult, usually difficult, sometimes difficult, usually easy, or always easy		1-5		A higher score indicates a higher level of health literacy		✓		✓		✓	
**eHealth literacy**														
	**eHealth literacy questionnaire (eHLQ; 7 of 7 domains)**													
		Using technology to process health information (5 items) Understanding health concepts and language (5 items) Ability to actively engage with digital services (5 items) Feel safe and in control (5 items) Motivated to engage with digital services (5 items) Access to digital services that work (6 items) Digital services that suit individual needs (4 items)	All items have the same response options: strongly disagree, disagree, agree, or strongly agree		1-4		A higher score indicates a higher level of eHealth literacy		✓		✓		✓	
**Health-related quality of life (HRQL)**														
	**RAND 12 (2 of 2 domains)**													
		Physical health composite (6 items)	All items have their individual scoring, and the physical composite score is based on the following 6 items: General health (excellent, very good, good, fair, or poor) Moderate activities, and Climb several flights of stairs, are scored on: limited a lot, limited a little, or not limited at all Accomplished less (physical), and 5. Limited in any kind of work, are scored on yes or no Pain interference (not at all, a little bit, moderately, quite a bit, or extremely)		0-100		A higher score reflects good physical health.		✓		✓		✓	
		Mental health composite-12 (6 items)	Accomplished less (emotional) (yes or no) The following items have the same scoring: (all of time, most of the time, a good bit of the time, some of the time, a little of the time, or none of the time) Did work less careful Calm and peaceful Energy Downhearted and blue Social limitations (time)		0-100		A higher score reflects good mental health.		✓		✓		✓	
**Service User Technology Acceptability Questionnaire (SUTAQ; intervention arm only)**														
	**SUTAQ (5 of 5 domains)**													
		Perceived benefit (9 items) Kit as a substitute (3 items) Satisfaction (3 items)	All items have the same response options: completely agree, moderately agree, mildly agree, mildly disagree, moderately disagree, or strongly disagree		1-6		A higher score reflects higher satisfaction.				✓		✓	
		Privacy and discomfort (4 items) Care personnel concerns (3 items)	All items have the same response options: completely agree, moderately agree, mildly agree, mildly disagree, moderately disagree, or strongly disagree		1-6		Reversed scale, thus higher score reflects a higher concern.				✓		✓	
**Satisfaction in general**														
	Satisfaction with the outpatient service in general (3 items)		All items have their individual scoring. Satisfaction with treatment (very satisfied, satisfied, neither satisfied nor dissatisfied, dissatisfied, or very dissatisfied) 2. Benefit from treatment (much better, somewhat better, stayed the same, somewhat worse, or much worse)		1-6		A higher score reflects higher satisfaction.		✓		✓		✓	
	Satisfaction with the outpatient service in general (3 items)		Safer with digital outpatient services (Yes; Feel free to elaborate/ No; Feel free to elaborate)		Y/N		Yes–safer		✓		✓		✓	
**COVID-19**														
	Perceived safety related to the COVID-19 pandemic (2 items)		Previously infected (yes or no)		Y/N		Yes–previously infected		✓		✓		✓	
	Perceived safety related to the COVID-19 pandemic (2 items)		Fear of COVID-19 (never, seldom, sometimes, often, or almost always)		1-5		A higher score reflects more fear		✓		✓		✓	

**Table 5:** Background variables and clinical outcomes.

Variables			Response options		Time point				
					T0		T1		T2
**Background variables**									
	Gender	Male, female, or unwilling to answer		✓					
	Marital status	Unmarried, married, cohabitant, widow or widower, divorced, separated, registered partner, divorced partner, or surviving partner		✓					
	Education	No education or preschool education, elementary school, high school without diploma, high school with diploma, bachelor’s degree or university or college lower level, master’s degree or university or college higher level, or PhD or researchers training		✓					
	Employment status	Full-time, part-time, home-employed, pursuing further education, unemployed, disabled, or retired		✓					
	Lifestyle habits (3 items)	(1) Smoking (yes or no), (2) snuff (yes or no), and (3) alcohol consumption (yes, no, and if yes, how often)		✓					
	Digital skills (3 items)	(1) Use of smartphone (yes or no), (2) use of tablet (yes or no), and (3) use of computer (yes or no)		✓					
	Use of mobile health apps	Yes or no–if yes, specify which apps		✓		✓		✓	
**Clinical variables (from the medical record)**									
	Primary diagnosis			✓					
	Time since diagnosis or start of condition	Duration of condition		✓					
	Medication	Current treatment and any changes		✓		✓		✓	
	Comorbidities	Number of comorbidities		✓					
**Use of health care resources (from the medical record)**									
	Contact with the outpatient clinic	Contact type (physical consultation, video consultation, telephone call, or other), planned or acute, and number of each						✓	
	Use of the digital service (dose of intervention)	Contact type (PROM^a^ response, task, chat, video, or other) and the number of each						✓	

^a^PROM: patient-reported outcome measure.

#### Health Literacy

The HLQ is a multidimensional measure of health literacy, with 44 questions across 9 domains. The HLQ is validated for adults using various modes of administration, including computer-based [51,52]. The HLQ was developed in Australia, translated into Norwegian, and used in studies on chronic diseases [51,52]. To reduce overlap with the domains of the eHealth literacy questionnaire (eHLQ), 5 of the HLQ domains (domain 1,2,3,6, and 9) that represent all 3 levels of the Nutbeam model have been included in this study [14]. Domains 2 and 9 are at the basic level; domains 1, 3, and 6 are at the communicative level, while domain 3 is at the critical level of health literacy [51].

#### eHealth Literacy

The eHLQ is multidimensional with the same origin as the HLQ, assessing people’s interactions with digital services based on the eHealth literacy framework [53]. The eHLQ contains 35 questions over 7 domains, and the full questionnaire is applied in this study. Although the eHLQ is translated into Norwegian and used in Norway, it is not yet validated. Validation in Denmark shows good psychometric properties [53].

#### HRQL

To assess HRQL, the patients fill out the RAND-12 [54,55], an abbreviated version of its predecessor, the SF-36. The 12 items are summarized into 1 physical and 1 mental health composite. The RAND-12 is validated in Norwegian [56].

#### Acceptability of the Digital Intervention

To assess acceptability among the participants assigned to the intervention arm, the patients fill out the service user technology acceptability questionnaire (SUTAQ). Acceptability refers to whether a system is good enough to satisfy users’ needs and requirements. The SUTAQ also assesses the importance of having contact with health care workers and whether this may affect patients’ acceptance [57,58]. The SUTAQ has 22 items across 5 domains. The participants are instructed to keep the digital outpatient service intervention in mind when responding. The SUTAQ has been translated into Norwegian, psychometrically tested [58], and is used in Norway for patients with type 1 and type 2 diabetes.

### Qualitative Evaluation Measures

Individual qualitative interviews will be conducted following the same structure for patients in the intervention group and the health care workers. The interviews will explore the interviewees’ perceived satisfaction with the digital outpatient service, and security in and beyond pandemic situations. The semistructured guide has been developed by the research team, inspired by topics on innovation assessment [59] and is added as a Multimedia Appendix 4. The aim of the qualitative interviews is to gain in-depth knowledge about the components of the intervention, how they are used, and whether and how they are perceived as useful. The interviews are also intended to obtain in-depth knowledge about how the digital intervention differs from traditional consultations and how these differences are perceived. In line with the primary outcome of health literacy obtained from the quantitative measures, any experiences of improved or otherwise affected health literacy will be investigated. Accordingly, the same guide will be applied to health care workers, tailored to their profession.

### Sample Size

An *a priori* sample size calculation was conducted to estimate the number of patients from all departments necessary to recruit based on changes from the baseline to the 6-month follow-up in the generic primary outcome “understanding health information well enough to know what to do” (HLQ domain 9) [51]. Previous research was summarized to find the most fitting SD. No similar studies were identified. Thus, an SD of 0.6 was applied in this study’s calculations based on 3 identified studies: one study on patients with epilepsy, reporting an SD of 0.77 after 18 months of follow-up [60], and studies reporting an SD of 0.42 in patients after kidney transplant [61] and an SD of 0.025 in the Danish norm data [62]. With no previous description of a minimally important difference for this exact domain, a 10% change on this scale of 1-5 was calculated, ending in a 0.5-unit difference between the groups. The analysis was based on a power of 0.90, an SD of 0.6 from the outcome measure, and a 2-sided significance test. The effect size was a 0.5-unit difference between the groups on a scale of 1-5, with a 20% dropout and an opportunity to perform controlled analyses. With a 1:2 recruitment ratio, this study must have a minimum of 55 participants in the control group and 110 participants in the intervention group. This is equivalent to a total sample size of 165 participants. When divided among the 5 departments, each department must recruit 33 participants. However, the total sample size is a shared goal. The intention was a 1:1 allocation of participants; however, to provide a more efficient recruitment when recruitment was slow, a statistician was consulted, suggesting an alternation in the allocation to ensure a sufficient sample size in favor of both groups.

For the qualitative interviews, this study needs a sample of 12-15 patients from the intervention group [63] and 12-15 health care workers or stakeholders that have experience with the tailoring of, or patient follow-up, using the digital outpatient intervention.

### Analysis

#### Statistical Analysis

The baseline and follow-up variables will be presented descriptively. Continuous variables will be analyzed using the mean and the SD for normally distributed data and the median and the range if the data are skewed. Categorical data will be presented as counts and percentages. The mean change will be estimated by subtracting the baseline score from the follow-up score, both at 3 months and 6 months. Differences in mean changes in short-term and long-term variables will be modeled using a one-way ANOVA. To adjust for possible confounders, logistic regression models will include age, gender, education, and hospital department. Missing data will be handled according to the syntax and protocol of the standardized instruments, such as HLQ, eHLQ, SUTAQ, and RAND-12. The missing data in terms of dropout has been accounted for in the power analysis.

#### Qualitative Analysis

Individual interviews with patients and health care workers will be audio-recorded, transcribed verbatim, and analyzed using thematic analysis [64], with the following steps: (1) familiarize with the data; (2) generate initial codes; (3) search for themes; (4) review themes; (5) define and name the themes; and (6) produce the report. Examples of data extracts and codes will be presented alongside the final thematic analysis results. Patient interviews will be analyzed by 2 researchers (HH and EF), while health care worker interviews will be analyzed by HH, EF, and an associated member of the research team. Any conflicts during the analysis will be resolved through discussion as a natural part of the analysis process. Preliminary findings will be presented to the project group to add reflections and nuances not already addressed.

### Data Security

Digital consent and digital responses to the questionnaires are collected using a service for sensitive data developed at the University of Oslo. The service for sensitive data is designed for storing and processing sensitive data in compliance with the Norwegian “Personal Data Act” and “Health Research Act.” The questionnaires are sent to the participants through the pretty good privacy-encrypted version of the University of Oslo web-questionnaire service “Nettskjema” which demands a governmental ID portal for login and allows secure data harvesting. A personal, secure link to the follow-up questionnaires is sent to the participants’ email or mobile phone, with 1 reminder after 6-7 days in the case of unanswered questionnaires, followed by a phone call if they still have not filled out the pending questionnaire after 1 more week.

### Ethics Approval

The regional ethical committee (REC) in Norway prereviewed the protocol and judged the project as outside its mandate according to the Norwegian Health Research Act (REC south-east reference number 252051). The project was reviewed by the institutional data protection officer at UNN regarding the cancer department (reference number 2021/4942) and the data protection officer at OUH regarding the remaining departments (reference number 21/06826); both granted approval.

## Results

### Trial Status

Recruitment started in September 2021, and as of December 14, 2022, a total of 55 patients had been enrolled in the control group, and 101 patients had been enrolled in the intervention group. It is expected that the recruitment will be completed by the end of December 2022 and that the 6-month follow-up for all participants will end by June 2023. Qualitative interviews will be conducted successively as the participants complete their 6-month assessments. All study results are expected to be ready by the end of 2023.

### Deviations From the First Registration in Clinical Trials

Initially, this study was a 2-arm trial with a 1:1 recruitment ratio. Recruitment was planned in 4 departments, and the department of pain management aimed at recruiting both patients with chronic pain as well as acute postoperative pain. However, due to unforeseen challenges, the recruitment of patients with postoperative pain was terminated in March 2022. Thus, a new power analysis was performed with reduced heterogeneity. Additionally, the follow-up period was shortened from 12 months to 6 months due to the lengthy process of the risk and vulnerability assessment before the use of the digital outpatient service in the hospital setting, which had not been done before this study.

## Discussion

Evidence regarding the need for multicomponent digital outpatient services and their possible effects on outcomes in a real-life setting remains scarce [12,16]. Therefore, this study will likely provide valuable knowledge as it aims to assess the impact and acceptability of digital outpatient services and their effects on health literacy, HRQL, and the acceptability of the digital intervention. The analyses will also address how the digital outpatient intervention is used. In-depth perspectives gathered from the qualitative interviews with patients and health care workers will add value to the qualitative findings. This study will provide important knowledge about the applicability and effects of digital health care services. As a result, patients and health care workers will gain a new, evidence-based understanding of whether and how digital tools may be used in clinical care.

The described intervention will be delivered through a multicomponent digital platform (Dignio) that has been implemented and studied in various health care settings in recent years. This way, digital usability is considered ready for evaluation on a larger scale in specialized outpatient care services. By collaborating with the clinical environment and the patient’s journey to identify the best potential for the digital intervention, the relevance of the intervention is increased. Likewise, complementing the digital platform with PROMs and clinical measures based on the expected needs according to the patient’s diagnosis will likely facilitate use among patients and health care workers. Together, these will add value to this study’s evaluation of health literacy, HRQL, and the acceptability of the intervention.

This intervention will support and enhance patients’ understanding of health information well enough to know what to do, make them feel understood and supported by health care workers, ensure that they have sufficient health information, support their feeling of actively managing their health, and improve their ability to interact with health care workers. These actions are directly transferable to the HLQ and this study’s outcome domains [51]. Additionally, the intervention will support patients’ use and understanding of digital solutions for health and thus their engagement, as well as ensure their access to digital solutions that work, suit their needs, and keep their health data secure, all of which are transferable to digital health literacy [12,53]. Altogether, this intervention will target and measure the domains of health literacy skills at the basic, communicative, and critical levels [14]. This study will also consider factors that are affected by external factors in health care services and the internal factors held by the patient. As this intervention will be individualized according to each patient’s needs, it can facilitate health actions and lead to an increase in health literacy.

This study describes how the digital outpatient service intervention will be explored in a multicenter, nonrandomized controlled trial with 2 treatment arms and a 6-month follow-up. The strengths of the current trial include its focus on patient-reported health literacy in a digital health intervention that targets a diverse sample of outpatients. Previous interventions have tended to focus on a single component, target a very specific sample, and primarily use clinical end points. Whether or not a patient chooses to use a digital intervention perhaps relies more on their health literacy and motivation to self-monitor, and a positive change in clinical end points may primarily be perceived as a potential result of their health literacy levels and self-monitoring. Moreover, the multimethod design inspired by the MAST [50] will provide quantitative data on the effects of the intervention on health literacy and in-depth qualitative knowledge on the acceptability of the intervention among patients and health care workers. Overall, the proposed trial will evaluate the effects while providing an understanding that can facilitate lasting changes in how outpatient specialty health services use digital solutions.

The proposed trial is designed as a nonrandomized trial, which inherently includes some risk of bias. Thus, differences may occur between the control group and the intervention group. The groups will therefore be compared at baseline to detect any imbalances that would need handling in the statistical analysis. Changes in outcome measures will be calculated based on the change from baseline to follow-up, thus handling the fact that individuals might have a different baseline score. Further, we did not pilot the intervention within each group before the full trial, which could have revealed weaknesses relevant to the full trial. Lastly, the outcomes measured by standardized questionnaires have not been used to screen for any need for a more tailored intervention. That is, it could have been relevant to tailor the intervention based on the participants’ scores in health literacy or digital health literacy. Individual health care workers have individualized patient follow-up based on the patients’ needs, but not through systematic screening. Also, we do not have data on the occurrence of any comorbidities in the sample, which could have been a relevant confounder added to the already described ones. No patients were specifically involved in the development of this intervention or the design of this trial. However, the intervention toward patients with epilepsy was developed alongside user representatives before this study.

## Appendix

Multimedia Appendix 1 DignioPrevent screenshot.

Multimedia Appendix 2 MyDignio.

Multimedia Appendix 3 Patient journey maps from all departments.

Multimedia Appendix 4 Interview guides.

## Abbreviations

CE: Conformité Européenne
eHLQ: eHealth literacy questionnaire
HLQ: health literacy questionnaire
HRQL: health-related quality of life
ILD: interstitial lung disease
MAST: method for assessment of telemedicine
OUH: Oslo University Hospital
PROM: patient-reported outcome measure
REC: regional ethical committee
SPIRIT: Standard Protocol Items: Recommendations for Interventional Trials
SUTAQ: service user technology acceptability questionnaire
TiDiER: Template for Intervention Description and Replication
UNN: University Hospital of North Norway

## Data Availability

Data sharing is not applicable to this paper as no data sets were generated or analyzed during this study. Future data collected as part of the study described in this protocol will be made available from the corresponding author upon reasonable request.
