# Supplementary material for: Digital Outpatient Services for Adults: Development of an Intervention and Protocol for a Multicenter Non–Randomized Controlled Trial
Source: JMIR Res Protoc. 2023 Jul 10;12:e46649. doi: 10.2196/46649 (PMC10366969; doi:10.2196/46649)
Supplement: Multimedia Appendix 1 [file resprot_v12i1e46649_app1.docx]

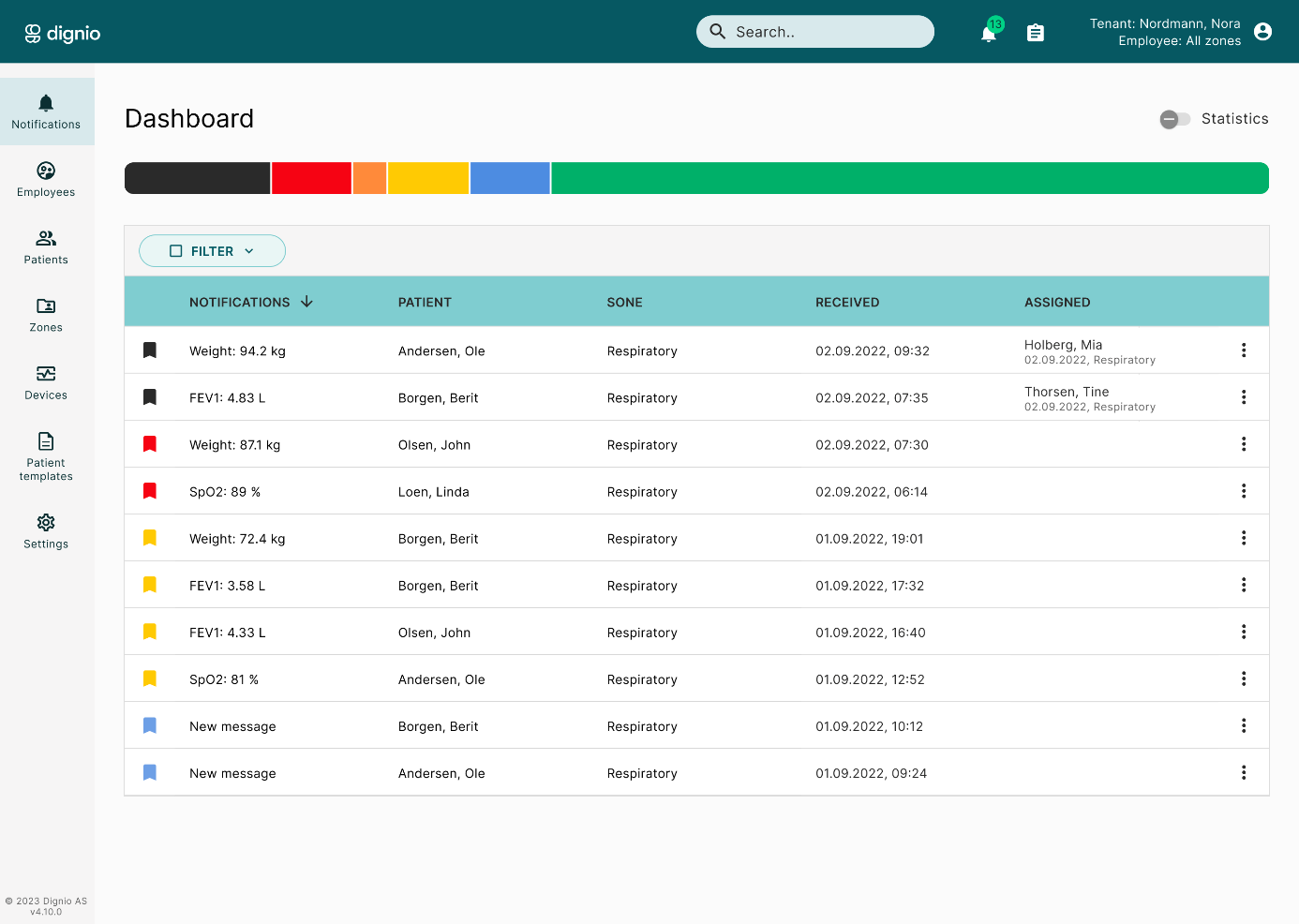


Appendix figure 3. Dashboard in DignioPrevent, as the healthcare workers see it. All names, dates and values are repoduced and do not contain real patient or healthcare worker data.
