# Supplementary material for: Digital Outpatient Services for Adults: Development of an Intervention and Protocol for a Multicenter Non–Randomized Controlled Trial
Source: JMIR Res Protoc. 2023 Jul 10;12:e46649. doi: 10.2196/46649 (PMC10366969; doi:10.2196/46649)

As-is: Patients receiving chemotherapy

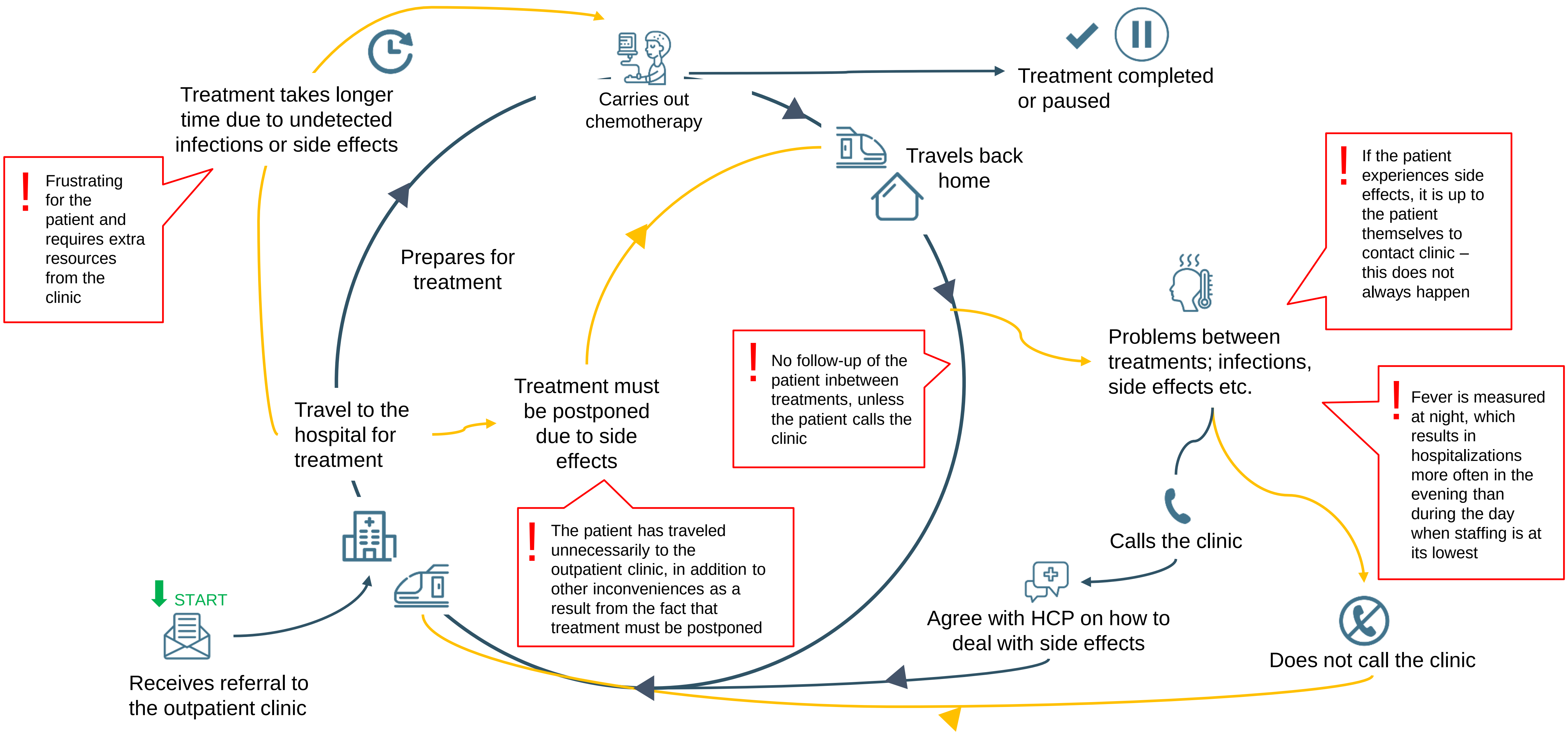

To-Be: Patients receiving chemotherapy

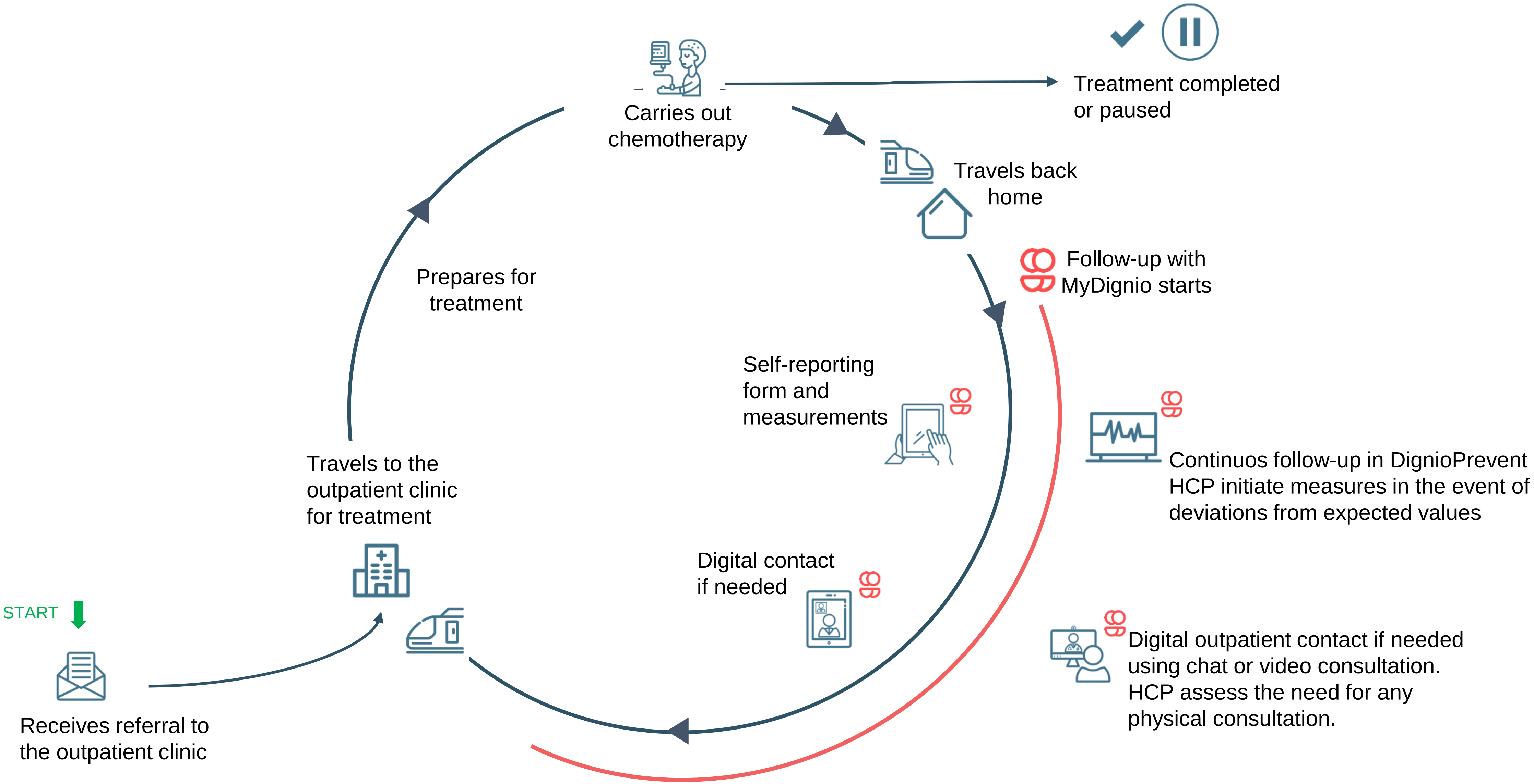

As-Is: Patients with epilepsy

Receives referral to the outpatient clinic

→ Travels to the outpatient clinic

→ Physical meeting at the outpatient clinic with a neurologist, gets Rx and information.

! Often the patient has to travel home and back between the first check-up and additional examinations as they cannot always be scheduled at the same time / day

→ Any additional examinations (e.g. EEG, MR)

→ Travels back home

↓ Receives a new appointment at the outpatient clinic in 4-6 weeks.

! This appointment is often postponed due to lack of capacity / waiting list at the outpatient clinic

Travels to hospital for a scheduled appointment at the outpatient clinic

← Physical meeting at the outpatient clinic with a neurologist or an epilepsy specialist nurse

← Receives a new appointment at an outpatient clinic in 1-2 years.

! Hours at the outpatient clinic are set up according to the calendar and not according to the patient's actual needs

← Travels back home

! There is little follow-up of the patients between the annual check-ups (every other year)

← Travel to the outpatient clinic for annual check-ups at the outpatient clinic in 1-2 years

! Some patients travel unnecessarily for control, as there is not always a clinical need for control

To-Be: Patients with epilepsy

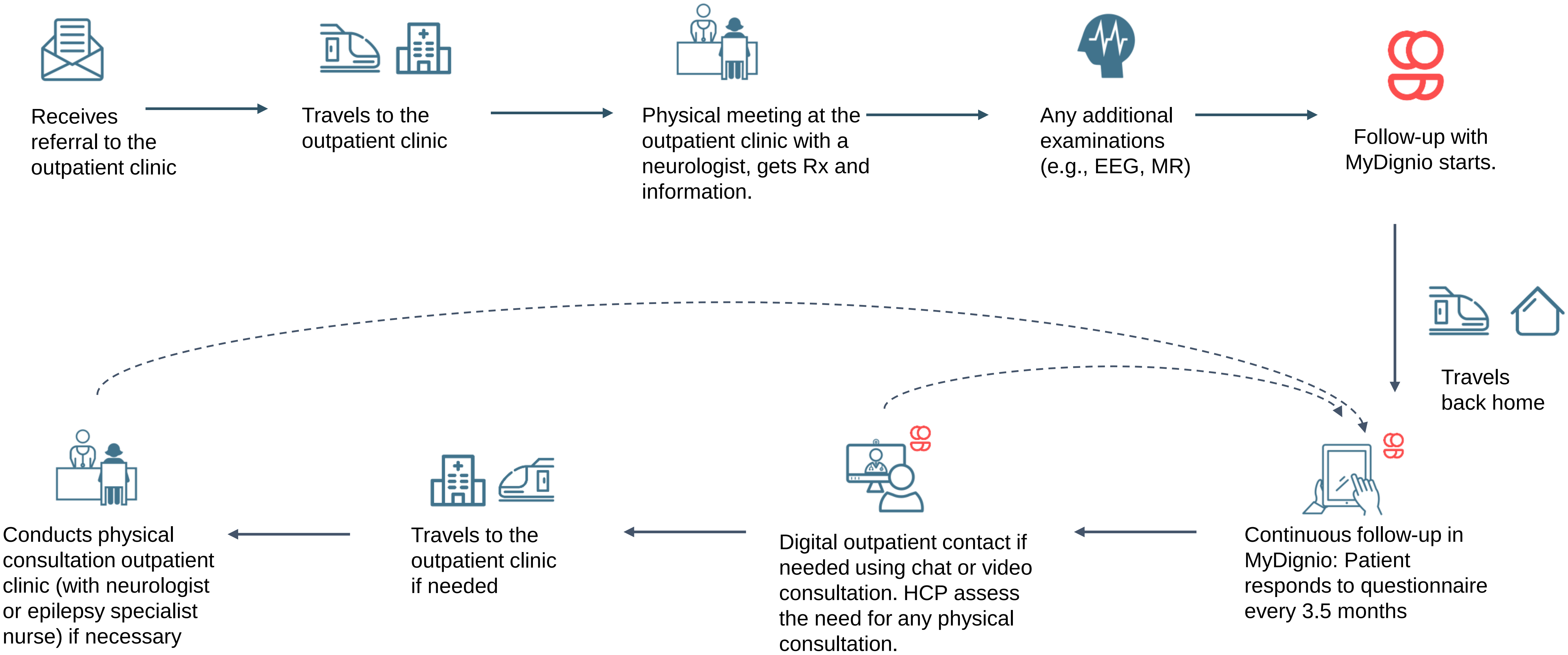

As-Is: Drug conversion for patients with complicated pain

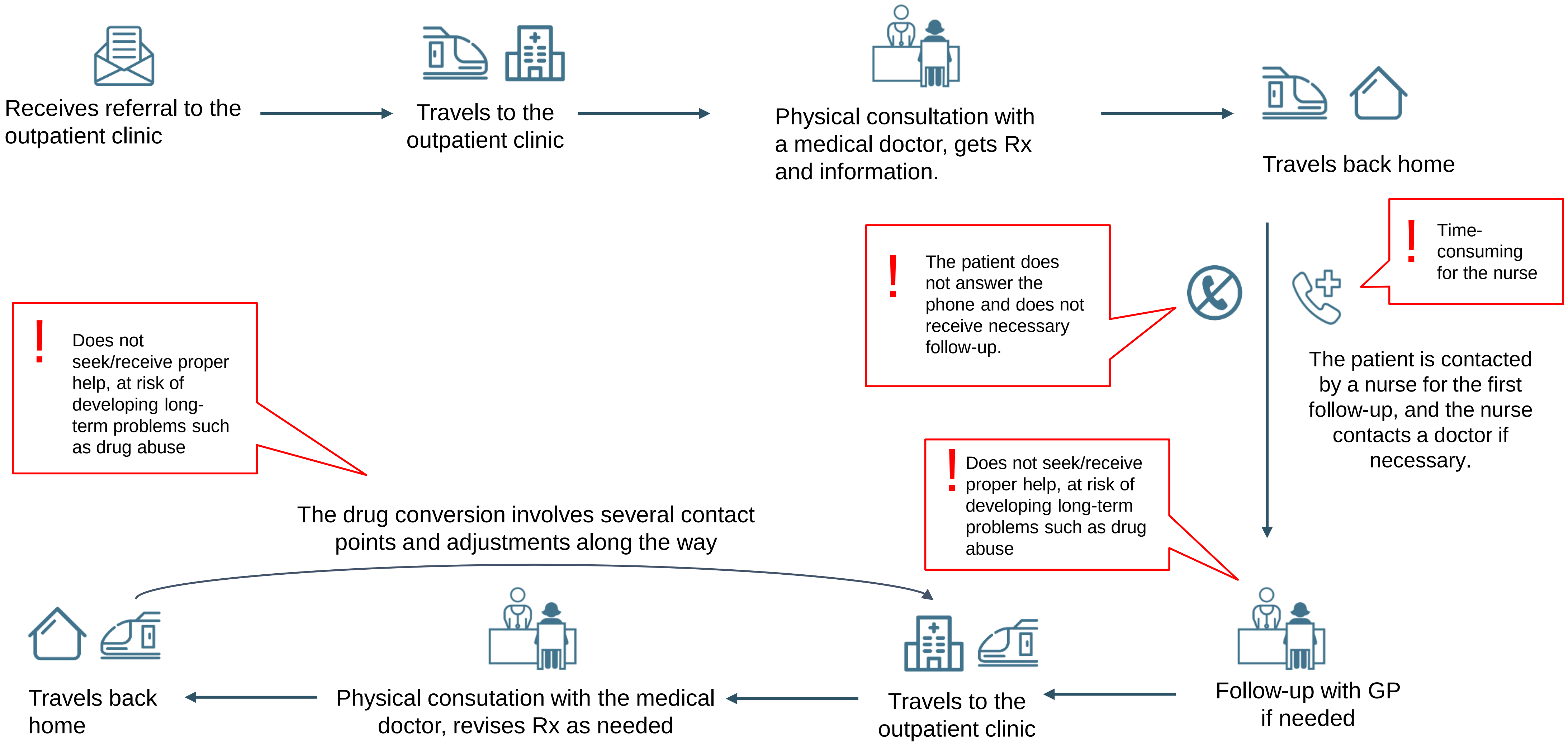

To-Be: Drug conversion for patients with complicated pain

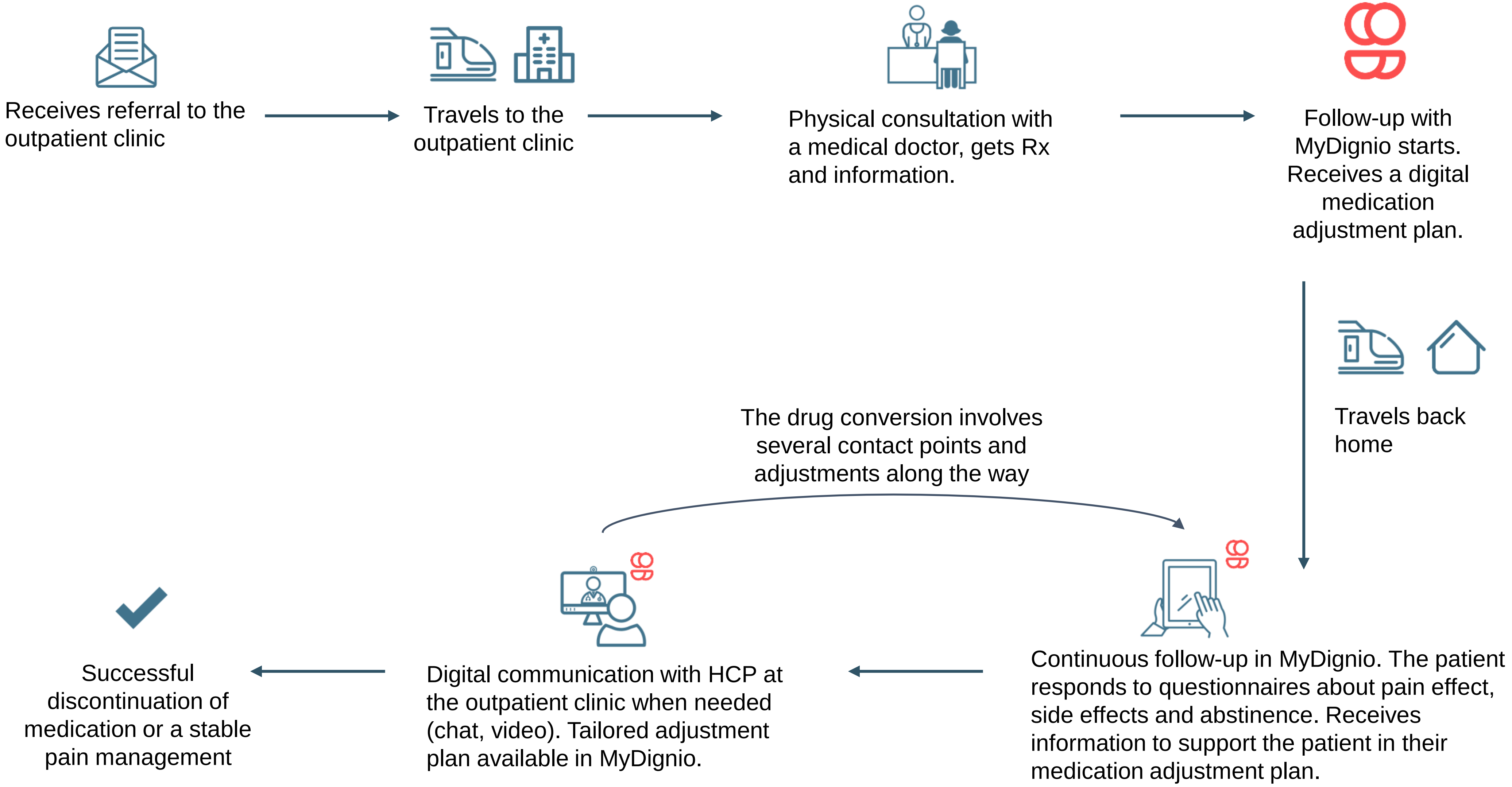

As-Is: Patients with pulmonary fibrosis

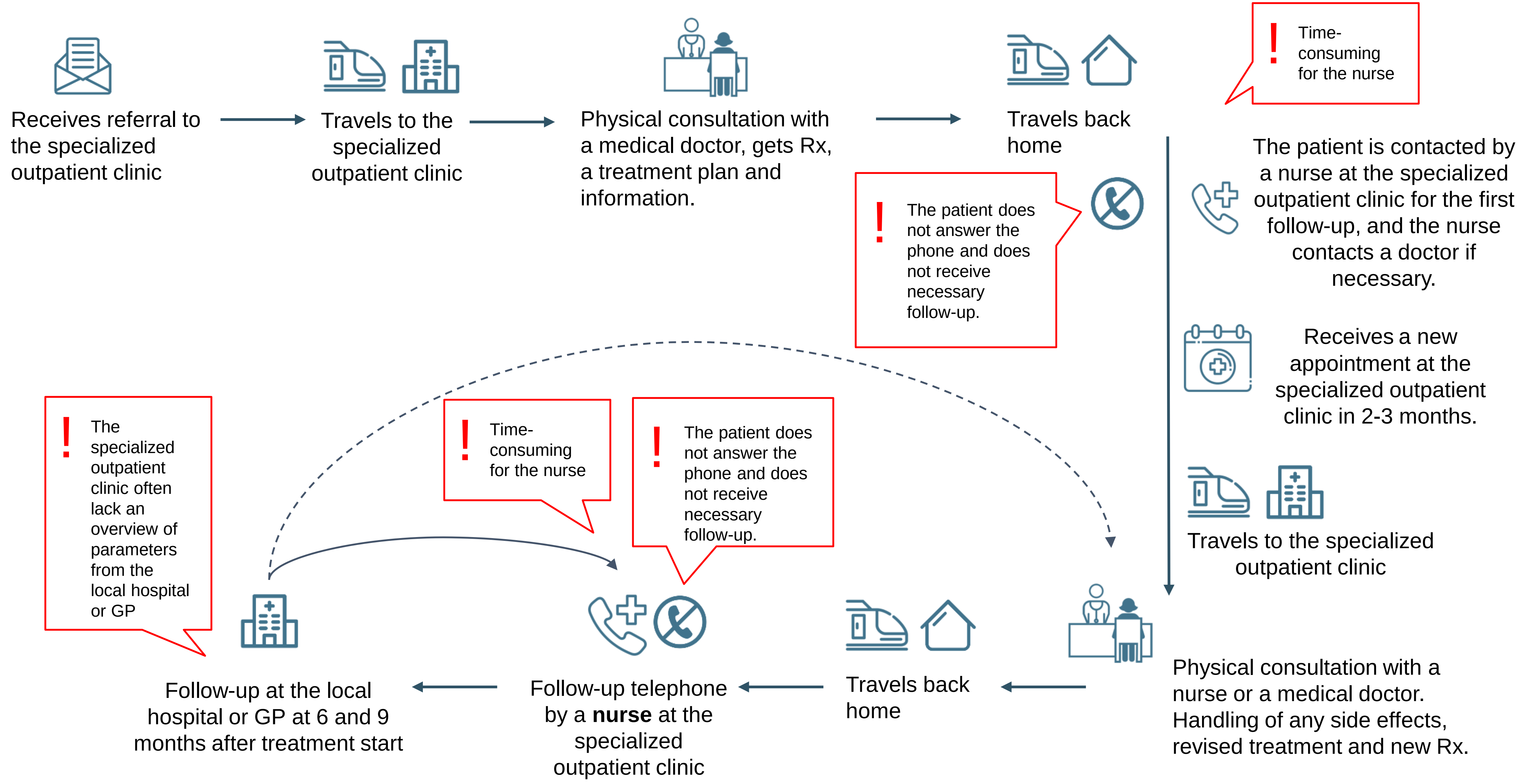

To-Be: Patients with pulmonary fibrosis

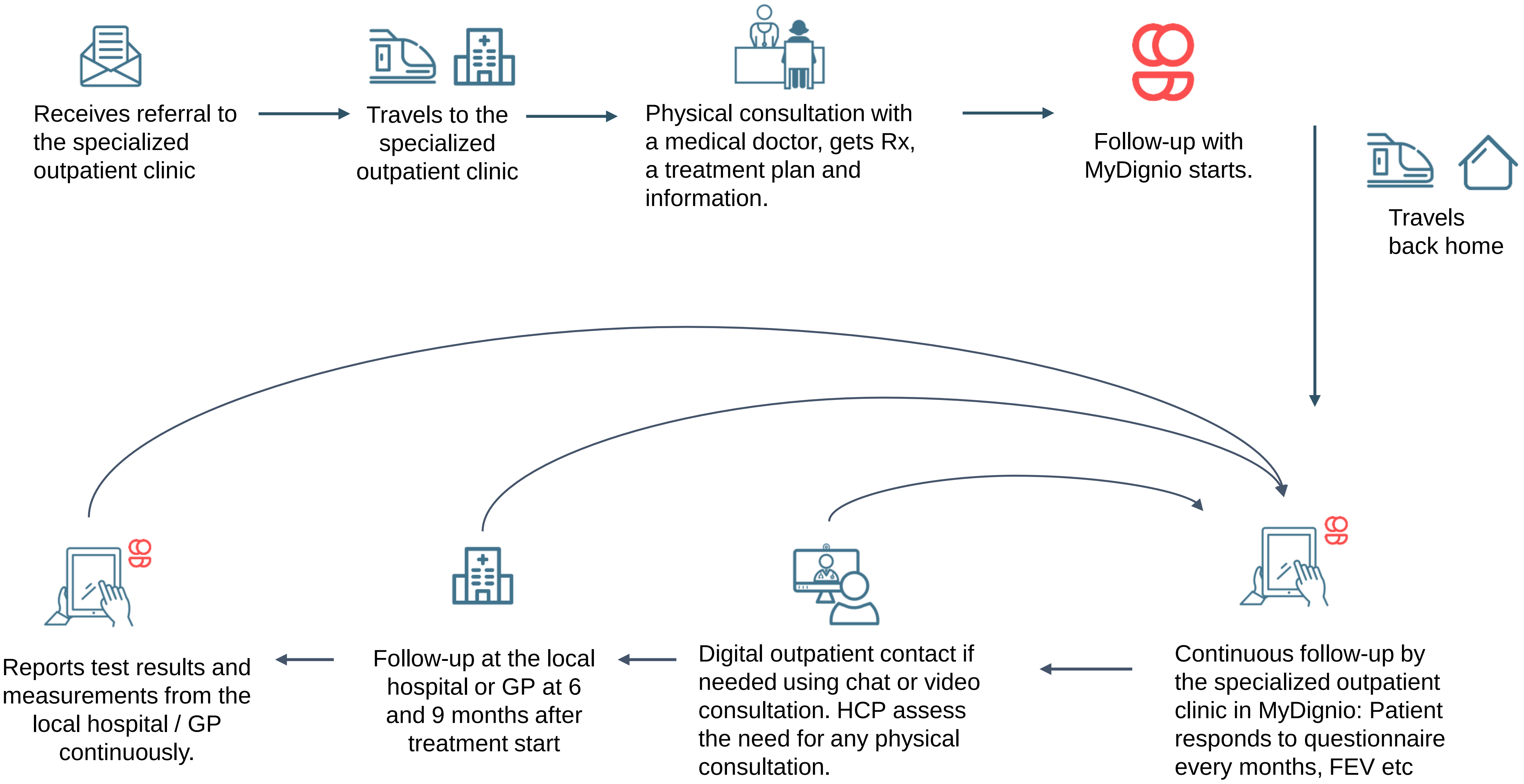

Supplement: Multimedia Appendix 3 [file resprot_v12i1e46649_app3.pdf]
